# Disrupted Causal Connectivity in Mesial Temporal Lobe Epilepsy

**DOI:** 10.1371/journal.pone.0063183

**Published:** 2013-05-16

**Authors:** Gong-Jun Ji, Zhiqiang Zhang, Han Zhang, Jue Wang, Dong-Qiang Liu, Yu-Feng Zang, Wei Liao, Guangming Lu

**Affiliations:** 1 National Key Laboratory of Cognitive Neuroscience and Learning, School of Brian and Cognitive Sciences, Beijing Normal University, Beijing, China; 2 Department of Medical Imaging, Jinling Hospital, Nanjing University School of Medicine, Nanjing, Jiangsu, China; 3 Center for Cognition and Brain Disorders and the Affiliated Hospital, Hangzhou Normal University, Hangzhou, Zhejiang, China; 4 Zhejiang Key Laboratory for Research in Assessment of Cognitive Impairments, Hangzhou, Zhejiang, China; Cuban Neuroscience Center, Cuba

## Abstract

Although mesial temporal lobe epilepsy (mTLE) is characterized by the pathological changes in mesial temporal lobe, function alteration was also found in extratemporal regions. Our aim is to investigate the information flow between the epileptogenic zone (EZ) and other brain regions. Resting-state functional magnetic resonance imaging (RS-fMRI) data were recorded from 23 patients with left mTLE and matched controls. We first identified the potential EZ using the amplitude of low-frequency fluctuation (ALFF) of RS-fMRI signal, then performed voxel-wise Granger causality analysis between EZ and the whole brain. Relative to controls, patients demonstrated decreased driving effect from EZ to thalamus and basal ganglia, and increased feedback. Additionally, we found an altered causal relation between EZ and cortical networks (default mode network, limbic system, visual network and executive control network). The influence from EZ to right precuneus and brainstem negatively correlated with disease duration, whereas that from the right hippocampus, fusiform cortex, and lentiform nucleus to EZ showed positive correlation. These findings demonstrate widespread brain regions showing abnormal functional interaction with EZ. In addition, increased ALFF in EZ was positively correlated with the increased driving effect on EZ in patients, but not in controls. This finding suggests that the initiation of epileptic activity depends not only on EZ itself, but also on the activity emerging in large-scale macroscopic brain networks. Overall, this study suggests that the causal topological organization is disrupted in mTLE, providing valuable information to understand the pathophysiology of this disorder.

## Introduction

Mesial temporal lobe epilepsy (mTLE) is a common epileptic syndrome [1], [2]. The mesial temporal lobe (mTL) structure is conventionally regarded to be responsible for generation of epileptic activity [3]. Recently, the technological development of resting-state functional magnetic resonance imaging (RS-fMRI) facilitates the identification of the abnormal intrinsic brain activity in patients with mTLE [4].

A number of RS-fMRI studies have found that, the abnormality of intrinsic activity is not restricted to mTL, and could be found in anatomically distant brain regions in mTLE patients. Both increased and decreased local activity can be observed in extratemporal regions using general linear model on simultaneously electroencephalograph (EEG)-fMRI data [5]. This technique, however, is still challenged for epilepsy study. One reason is the insensitivity of scalp EEG to detect discharges from a small cortical area (<10 cm^2^) or deep brain structures [6]. Another is the variability of hemodynamic response function, which is hard to be specified according to each subjects or discharges [7]. An alternative analysis strategy is the data-driven approach. Using temporal clustering analysis, Morgan et al. [8] found positive blood oxygenation level dependent (BOLD) fluctuations in temporal lobes and default-mode regions in temporal lobe epilepsy. In addition, a novel method as regional homogeneity (ReHo), that measured the temporal synchronization of the BOLD signal from neighboring voxels, has been used to study mTLE. Mankinen et al. [9] found ReHo increased in the posterior cingulate gyrus and mTL, and decreased in the cerebellum. More recently, using amplitude of low-frequency fluctuation (ALFF), Zhang et al. [10] found increased ALFF in the mTL. The ALFF measures the magnitude of the spontaneous BOLD signal, and it has been suggested to be associated with local neuronal activity [11]–[13]. Moreover, the ALFF was positively correlated with the number of epileptic discharges in mTLE [10], which suggests that the increased ALFF may reflect the epileptic activity. Thus, ALFF may be a complementary approach to EEG-fMRI studies to localize the epileptogenic zone (EZ) in mTLE [10].

Regarding epilepsy as a network disorder [14], [15], the investigation of functional synchronization change is critical to understand the pathophysiological mechanism of mTLE. Functional integration is not only used to observe the impairments in mTL associated network [16]–[18], but also in other functional networks, such default mode network (DMN) [19], [20], attention network [21], perceptual network [22], limbic system [17] and the whole brain network architecture [23]. The analytic methods in these studies, however, ignored the direction of information flow between brain regions, which is crucial to understand the seizure propagation from the EZ to other brain regions.

Recently, in order to characterize the abnormal information flow, some approaches have been used in epilepsy patients or experimental protocols, such as non-linear regression [24]–[28], dynamic causal modeling [29]–[31] and Granger causality analysis (GCA) [32]–[35]. GCA has been proved helpful to identify the direction of seizure propagation [35], [36]. In a region-of-interest (ROI) based research, Morgan et al. [34] performed GCA between bilateral hippocampus in mTLE. They found that, the hippocampus contralateral to EZ exerted more causal influence over the ipsilateral hippocampus, which is helpful to understand the functional development of epileptic networks. Most previous GCA studies are based on the F-test for the residual in multi-regression model [34], [36]. Because the F value is always non-negative, it can only detect the positive influence between brain areas. However, both positive and negative causal influences are essential to the maintaining of normal brain function, and the imbalance between them is a fundamental change in epilepsy [37]–[39]. Recently, Chen et al. used signed regression coefficient β instead of F value to estimate Granger influence [33]. A positive value of β may indicate positive influence and a negative β may indicate inhibitory influence or negative feedback. Hamilton et al. [40] applied this method to major depressive disorder, and found both increased excitatory and inhibitory effect in paitents, which advanced the neural theory of depression.

In the current study, we employ ALFF to identify EZ of patients and characterize the change of its causal relation with whole brain regions. Thalamus and basal ganglia (BG) are critical nodes in the epileptic network of mTLE [25], [41], [42]. Thus, we predict that their causal relations with EZ are altered in patients. Moreover, we examine whether the local activity in EZ is related to the abnormal driving effect on it.

## Results

### Between-group analysis of ALFF

**Figure 1:**
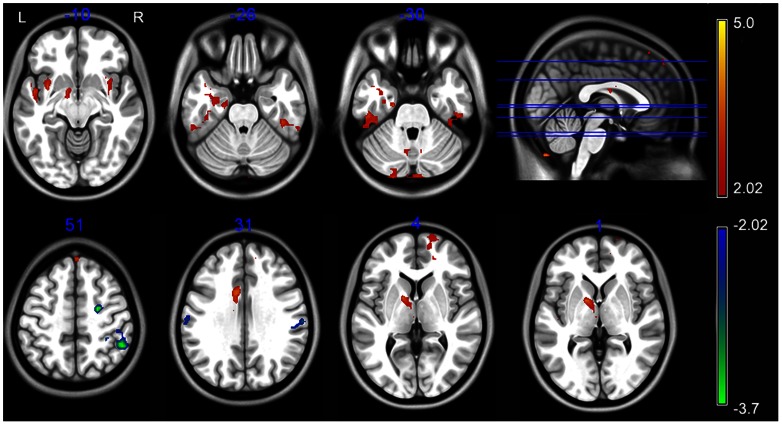
Regions showing abnormal amplitude of low-frequency fluctuation. The warm and cold colors represent higher and lower ALFF, respectively, in patients compared with controls (P<0.05, corrected). Color bar represents t-values.

As compared with the controls, the patients showed significantly increased ALFF in the left mTL, thalamus, pallidum nucleus, middle cingulate cortex, the bilateral inferior temporal gyrus, insular, cerebellum, and the right superior frontal gyrus (P<0.05, corrected) (Table 1 and Fig. 1). Brain regions showing decreased ALFF included the right premotor area and the bilateral supramarginal gyrus (P<0.05, corrected) (Table 1 and Fig. 1). The peak voxel within left mTL was located at MNI coordinate (−21, −15, −30).

**Table 1:** Regions showing abnormal amplitude of low-frequency fluctuation in patients.

Brain Region	BA	MNI (X Y Z)			Peak t-value	Cluster Size (mm^3^)
Inf. temporal gyrus R	20	42	−33	−24	3.54	3024
Inf. temporal gyrus L	20	−51	−21	−42	3.87	4023
mTL, insula L	20/36/13	−33	6	−9	4.05	8667
Insula R	13	39	−12	18	4.06	2052
Mid. cingulate cortex	24	−9	0	33	4.4	3267
Sup. frontal gyrus R	8	6	36	63	4.26	1512
Sup. frontal gyrus R	10	15	60	6	3.86	2673
Supramarginal gyrus R	40	51	−48	51	−3.72	5589
Supramarginal gyrus L	40	−45	−30	18	−3.29	1458
Premotor cortex R	6	24	−9	51	−3.66	1512
Thalamus, pallidum L	N/A	−15	3	3	4.13	5022
Cerebellar crus II R	N/A	0	−78	−51	4.41	7263
Cerebellar crus II L	N/A	−39	−60	−39	5.07	2214
Cerebellar crus II L	N/A	−21	−93	−30	3.12	2268
Cerebellar lobule VIII L	N/A	−3	−72	−54	3.85	10179

Abbreviation: BA = Brodmann's area; R = right side; L = left side; inf. = inferior; sup. = superior; Mid. = middle; mTL = mesial temporal lobe; MNI = Montreal Neurological Institute coordinates.

### Voxel-wise Granger causality analysis

#### Seed(EZ)-to-whole-brain

**Figure 2:**
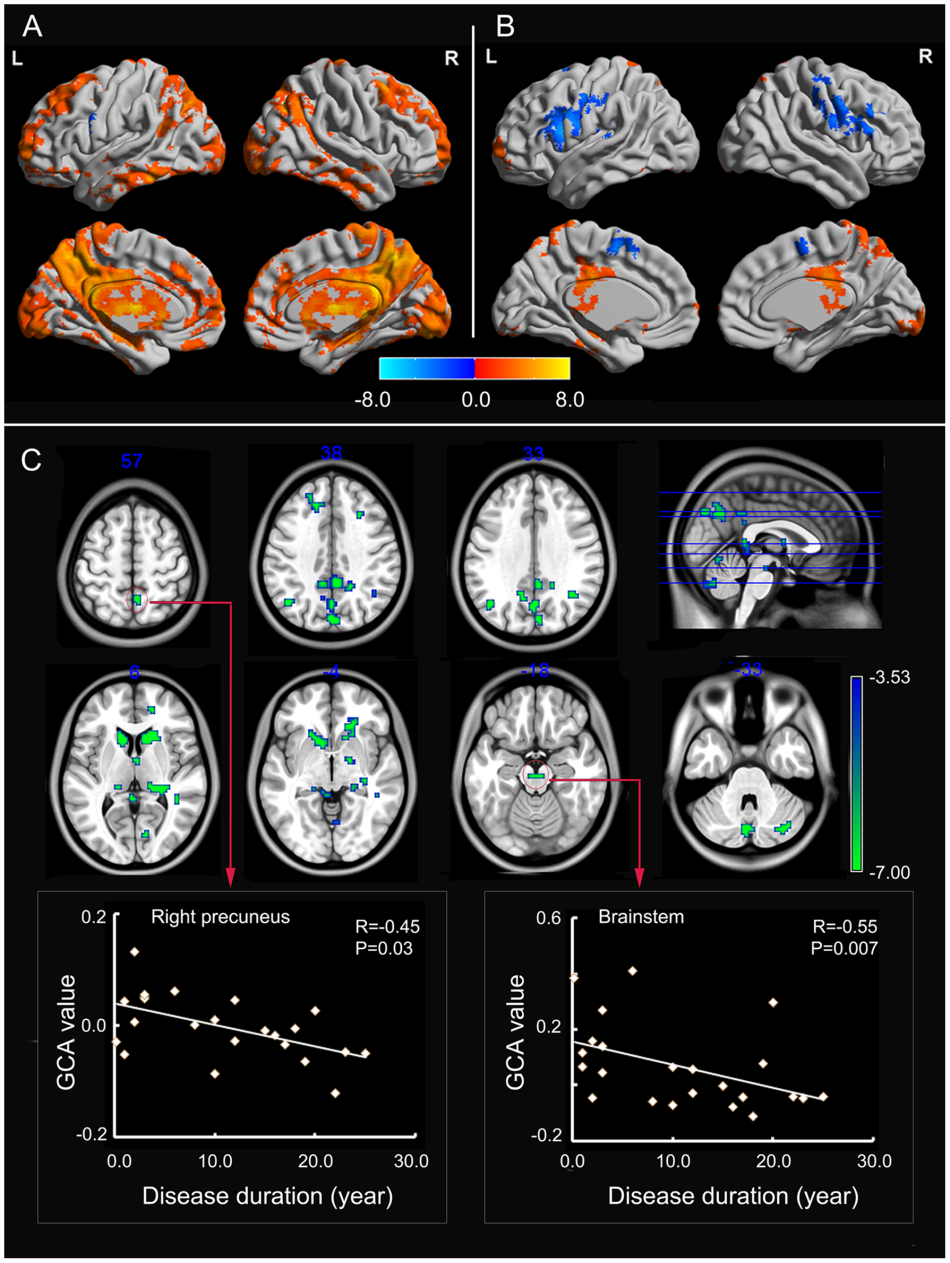
Granger causality analysis for seed(EZ)-to-whole-brain. (A) Regions showing significant causal effect with the seed in patients. (B) Regions showing significant causal effect with the seed in controls. Warm and cold colors indicate positive and negative causal effects, respectively. (C) Regions showing abnormal causal effect with the seed in patients compared with controls. The scatter-plot maps show the correlations between Granger causality value in corresponding clusters and disease duration. Color bar represents t-values.

Widespread cortical and subcortical structures were driven by the seed region in controls (Fig. 2A). The pattern in patients (Fig. 2B) was obviously distinct to that in controls.

Between-group analysis showed that the driving effect from EZ to subcortical structures decreased in patients (Fig. 2C, Table 2). In addition, cortical regions in the DMN were found with decreased causal effects, including the bilateral posterior cingulate cortex/precuneus (BA 31/7), angular gyrus (BA 39), and superior frontal gyrus (BA 32/9). Some regions in the visual network and cerebellum showed decreased causal effect with mTL in patients. The causal effect in precuneus (r = −0.45, P = 0.03) and brainstem (r = −0.55, P = 0.007) were negatively correlated with the disease duration (Fig. 2C).

**Table 2:** Regions showing abnormal causal effect with epileptogenic zone in patients (seed-to-whole-brain).

Brain Region	BA	MNI (X Y Z)			Peak t-value	Cluster Size (mm^3^)	mTLE	NC
**DMN**								
PCC	31	3	−42	36	−4.33	1539	0.85	5.68*
Ant. PCu. L	31	−12	−54	30	−4.27	270	−0.31	5.77*
Dor. PCu. R	7	6	−54	57	−3.95	243	2.11*	5.99*
Pos. PCu. L	7	0	−66	36	−4.08	729	0.39	4.71*
Ant. PCu. L	7/31	−15	−45	42	−4.21	972	0.73	5.39*
MPFC	11/32	18	48	3	−4.1	216	−1.18	5.52*
Angular gyrus L	39	−45	−66	33	−4.3	837	−1.39	4.85*
Angular gyrus R	39	36	−57	33	−3.67	216	0.24	5.38*
Sup. frontal gyrus R	9	30	24	39	−3.69	135	−1.11	4.70*
Sup. frontal gyrus L	9	−21	39	42	−4.68	324	−2.50*	3.96*
Sup. frontal gyrus L	32	−15	33	36	−4.25	270	−1.16	4.47*
**Visual Network**								
Calcarine R	17	6	−87	3	−3.87	189	−0.72	3.94*
Calcarine R	17	9	−78	3	−3.81	243	−2.27*	3.13*
Lingual gyrus R	18	12	−81	−12	−3.86	216	0.54	4.63*
Cuneus R	19	3	−84	36	−4.39	621	0.23	4.95*
**Subcortical Structures**								
Ant. thalamus L	N/A	0	−3	9	−4.15	162	1.12	6.47*
Pos. thalamus L	N/A	−12	−30	0	−4.46	1404	−1.11	4.45*
Pos. thalamus R	N/A	30	−33	12	−4.47	2376	0.55	6.36*
Caduate nucleus L	N/A	−12	15	0	−5.77	2619	−1.73	5.73*
Caudate nucleus R	N/A	15	12	15	−6.15	3834	−3.01*	5.40*
Lentiform nucleus R	N/A	18	−6	−6	−4.1	189	0.85	5.28*
Brainstem	N/A	3	−21	−18	−4.4	135	−0.24	5.21*
**Cerebellum**								
Vermis VIII	N/A	6	−66	−39	−4.54	243	−1.22	5.27*
Crus I R	N/A	33	−75	−33	−4.25	945	0.42	5.91*
Vermis VII	N/A	6	−69	−27	−4.74	1107	−0.68*	6.55*
Vermis VI	N/A	6	−66	−6	−3.98	243	0.34	4.77*

Abreviations: BA = Brodmann's area; R = right side; L = left side; sup. = superior; Ant. = anterior; Dor. = dorsal; PCC = posterior cingulate cortex; PCu. = precuneus; MPFC = medial prefrontal cortex; mTLE = mesial temporal lobe epilepsy; NC = normal controls; MNI = Montreal Neurological Institute coordinate.

The last two columns show the t-value of the corresponding peak voxel within patient and control group, respectively. Values with an asterisk show the mean causal effect of the corresponding cluster is significantly different from zero.

#### Whole-brain-to-seed(EZ)

**Figure 3:**
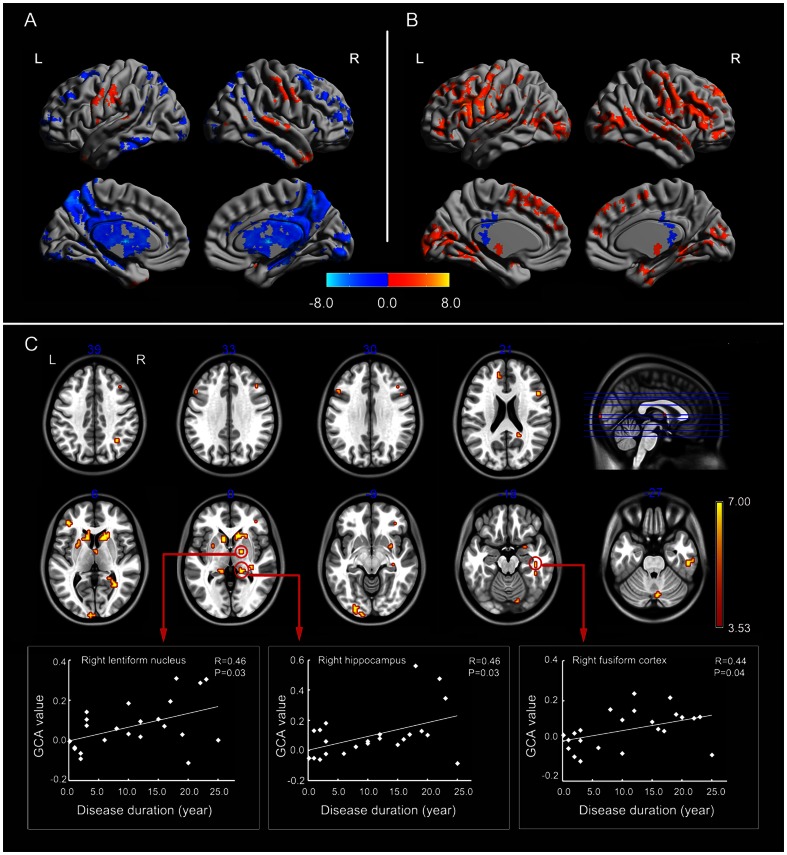
Granger causality analysis for whole-brain-to-seed(EZ). (A) Regions showing significant causal effect with the seed in patients. (B) Regions showing significant causal effect with the seed in controls. Warm and cold colors indicate positive and negative causal effects, respectively. (C) Regions showing abnormal causal effect with the seed in patients compared with controls. The scatter-plot maps show the correlations between Granger causality value in corresponding clusters and disease duration. Color bar represents t-values.

In controls, subcortical structures including thalamus and BG showed negative feedback to the seed region, and both a positive and negative driving effect were found from widespread cortical areas to EZ (Fig. 3A). The pattern in patients (Fig. 3B) was distinct from that in controls.

Between-group analysis showed the causal effect from a number of brain regions to EZ increased in patients (Table 3 and Fig. 3C) (P<0.05, corrected). In subcortical structures, the causal effect from thalamus and BG (bilateral caudate, left putamen, and right lentiform nucleus) to EZ increased in patients. In addition, there were widespread cortical regions showing increased driving effect to EZ. These regions were within the limbic system, ventral visual pathway, and executive control network (ECN). The limbic system included the bilateral hippocampus (BA 20/37/27), the right amygdala (BA 34), and the left dorsal anterior cingulate cortex (BA 32). The regions in ventral visual pathway included the left primary (BA 17)/secondary (BA 18) visual cortex, the bilateral associative visual cortex (BA 19), the right fusiform cortex (BA 37), and inferior temporal lobe (BA20). The ECN included the right angular gyrus (BA 39) and the bilateral inferior and middle frontal lobe (BA 9/44/45/46/47). The causal effect of right lentiform nucleus (r = 0.46, P = 0.03), right hippocampus (r = 0.46, P = 0.03), and right fusiform (r = 0.44, P = 0.04) positively correlated with disease duration (Fig. 3C).

**Table 3:** Regions showing abnormal causal effect with epileptogenic zone in patients (whole-brain-to-seed).

Brain Region	BA	MNI (X Y Z)			Peak t-value	Cluster Size (mm^3^)	mTLE	NC
**Limbic System**								
Hippocampus R	20	33	−27	−3	4.61	378	2.29*	−5.34*
Hippocampus L	37	−36	−36	−3	4.08	135	1.20	−5.32*
Amygdala R	34	27	3	−15	4.32	486	3.49*	−2.62*
Hippocampus L	27	−18	−33	0	3.72	135	1.66	−4.39*
Hippocampus R	27	15	−33	0	4.34	243	3.02*	−3.42*
ACC L	32	−12	45	24	3.83	162	2.74*	−2.77*
**Visual Network**								
Calcarine L	17	−6	−99	9	4.14	324	3.69*	−2.16*
Inf. occipital lobe L	18	−24	−90	−9	4.3	324	2.94*	−3.19*
Inf. occipital lobe L	18	−15	−102	−9	4.18	189	3.83*	−2.15*
Mid. occipital lobe L	19	−27	−84	15	3.76	189	2.49*	−2.98*
Calcarine R	19	30	−54	6	5.24	405	3.08*	−4.25*
Calcarine R	19	24	−45	6	4.1	162	2.69*	−3.11*
Fusiform R	37	42	−39	−15	4.69	135	2.41*	−4.34*
Fusiform R	37	42	−27	−18	4.07	162	1.33	−5.39*
Inf. temporal R	20	54	−24	−27	4.77	567	2.91*	−3.87*
**ECN**								
Sup. frontal sulcus R	46	21	36	24	4.26	216	1.90	−4.00*
Mid. frontal gyrus R	9	39	27	36	4	216	3.70*	−1.88
Sup. frontal sulcus L	46	−27	54	15	3.9	189	2.32*	−3.82*
Mid. frontal gyrus R	47	36	39	−3	4	270	2.28*	−3.43*
Pars triangularis R	46	39	36	15	4.1	189	3.67*	−1.83
Pars triangularis R	9	48	18	21	4.85	351	4.05*	−2.66*
Pars triangularis L	45	−42	39	6	4.43	324	4.41*	−1.16
Pars operculars L	44	−51	18	33	3.74	135	3.85*	−0.56
Angular R	40	33	−57	39	3.94	189	2.08*	−3.79*
**Subcortical Structures**								
Lentiform nucleus R	N/A	18	−3	−3	5.44	297	2.89*	−4.79*
Putamen nucleus L	N/A	−24	12	6	4.04	513	4.39*	−1.75
Caudate nucleus L	N/A	−9	15	0	5.07	1134	2.69*	−4.33*
Caudate nucleus R	N/A	12	18	12	6.49	2997	4.19*	−4.97*
Thalamus R	N/A	6	−3	3	3.89	162	1.37	−3.79*
**Cerebellum**								
Vemis VII	N/A	6	−72	−27	4.21	216	2.17*	−3.98*
Crus I R	N/A	24	−75	−21	3.74	135	0.68	−4.51*
Lobule VI R	N/A	15	−78	−15	4.01	135	2.52*	−3.17*

Abbreviation: BA = Brodmann's area; R = right side; L = left side; Sup. = superior; Mid. = middle; Inf. = inferior; mTLE = mesial temporal lobe epilepsy; NC = normal controls; MNI = Montreal Neurological Institute coordinates.

The last two columns show the t-value of the corresponding peak voxel within patient and control group, respectively. Values with an asterisk show the mean causal effect of the corresponding cluster is significantly different from zero.

**Figure 4:**
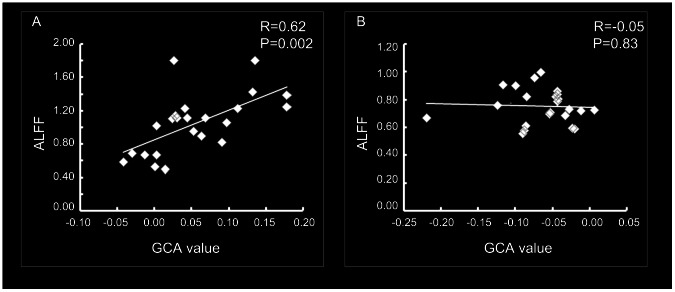
Correlation between the amplitude of low-frequency fluctuation (ALFF) of EZ and the abnormal driven effect on it in patients (A) and controls (B).

The averaged Granger causality value of the peak voxel in all the clusters showed abnormal driving effect to EZ, positively correlated with the ALFF value of EZ in the patient group (r = 0.62, P = 0.002) (Fig. 4A). This correlation was not significant in controls (r = −0.05, P = 0.83) (Fig. 4B).

## Discussion

In the present study, we identified EZ in mTL using ALFF and investigated its causal relation with other brain regions. The results indicated that there was a negative feedback circuit between the GCA seed and thalamus/BG in controls. Compared with controls, patients demonstrated decreased driving effect from EZ to thalamus/BG and increased feedback. In addition, we also found that some cortical regions (DMN, ECN, visual network, and limbic system) showed abnormal causal relation with EZ. These findings revealed widespread brain regions showing aberrant causal interaction with EZ. Furthermore, we found that the local activity level in EZ was positively correlated with the abnormal causal effect on EZ in patients. This correlation suggests that the highly spontaneous activity of EZ is partly contributed by the increased driving effect on EZ.

Using ALFF, we found that widespread brain regions showed increased local activity in patients. These findings are consistent with our previous study [10] and other multiple-modality studies [5], [37], [43]. ALFF is the magnitude of the spontaneous BOLD signal and may reflect the local neuronal activity [11]–[13]. Epilepsy is characterized by an excessive synchronization of neuronal activity, and it is conventionally thought that epileptic activity of mTLE originated from mTL. Thus, the left mTL region with increased ALFF is probably the EZ.

The structural and functional changes in subcortical structures have been widely reported in mTLE patients [10]. The thalamus has widespread functional and anatomical connections with the neocortex and limbic system [44], [45] and acts as an amplifier and synchronizer of epileptic activity [46], [47]. The BG showed an inhibitory influence on seizures in mTLE [48]. Recently, a resting-state fMRI study found alterations in functional connectivity between thalamus/BG and hippocampus [49]. In accord with these findings, we found the feedback circuit between EZ and subcortical structures altered in mTLE patients.

Using non-linear correlation, the causal connectivity between EZ and thalamus has been characterized by intracranial EEG [50]. But because of the possible discrepancy between fMRI signal and intracranial EEG in TLE [24], it is hardly to compare findings from these two modalities. Recently, a few fMRI studies investigated the causal connectivity changes in TLE patients [24], [31], [34]. Bettus et al. [24] found that, the causal connectivity between the regions affected by electrical epileptiform abnormalities was lower than that between non-affected areas during interictal state. Our finding (decreased causal effect from EZ to subcortical areas) is in line with this result. The exact reason for the causal connectivity abnormalities between EZ and subcortical areas remains unknown. It could be a result of the deafferentation from loss of efferent fibers from the epileptogenic area [51].

Abnormality of causal effect relating with EZ was also found in wide cortical structures, including the regions of DMN, limbic system, visual network and ECN. The decreased local or connectivity property of DMN has been reported in mTLE patients, indicating impairment of the default brain function [10], [19], [52]. The current study demonstrated decreased causal effect from EZ to DMN areas in the patients. This finding further implicated that the abnormal activity in DMN might be directly caused by epileptic activity from EZ. The decreased causal effects in the other cortical regions may suggest impairments of the corresponding functions. A resting-state fMRI study found decreased functional connectivity between limbic areas [17]. In line with this study, our Granger analysis found that EZ was driven by an abnormal positive effect from the limbic network. Abnormal functional connectivity within the visual system [22] and ECN [21] has been reported in our previous fMRI studies. Currently, we found their causal effect with epileptogenic area altered in patients as well. These abnormalities may related to the impairments of visual memory [53] and executive function [54] in mTLE patients.

In this study, we observed increased local activity within mTL ipsilateral to the epileptogenic side, which is probably EZ. According to Ding et al. [55], the power of one region is the sum of intrinsic power and causal power. In line with this point, a significant correlation was found between the activity levels and the causal influence across the DMN nodes [56]. Thus, the increased local activity of EZ was probably caused by the abnormal positive effects from other brain regions. To exam this hypothesis, we correlated the average GCA value in these regions and the ALFF of EZ across subjects. A positive correlation was found in patients, but not in controls. These results demonstrate that the high activity level of the epileptogenic zone is partly contributed by the abnormal driving effect from extratemporal regions, and it suggests the initiation of epileptic activity depends not only on EZ itself, but also on the activity emerging in large-scale macroscopic brain networks.

Coefficient-based GCA is a directed functional connectivity method [33]. One characteristic of this method is the ability to discriminate positive and negative effects between brain regions, which may correspond to excitatory and inhibitory effects, respectively [40]. Given that imbalance of excitatory and inhibitory effect is a fundamental change in epilepsy [37], [39], this characteristic of coefficient-based GCA has a special advantage for investigating the pathophysiological mechanism of mTLE. Although this method was applied to investigate epilepsy using resting-state fMRI data, several unanswered questions should be noted. Firstly, we used the bivariate, instead of multivariate GCA to investigate causal interaction between the EZ and the other voxels in the whole brain. Multivariate GCA [57] included all measured variables in the autoregressive model avoiding spurious causalities. However, it becomes ill-posed when we deal with high dimensional and short fMRI time series [58]. To deal with this problem in multivariate causality measures, some solutions were proposed, such as redundant and synergetic variables [59] and sparse regression techniques [60]. These methods deserve to be considered in the future work for whole brain voxel-wise GCA. Secondly, there are some considerations when applying Granger causality to fMRI data [61]–[63]. For example, GCA is a generic inferential procedure characterizing directed functional connectivity. It models dependency among observed responses. The indirect relation between haemodynamic responses and neuronal activity must be recognized [63]. Thirdly, the corresponding neuronal mechanism of Granger influence during resting state is not fully understood. To clarify the relation between positive/negative Granger influence and excitatory/inhibitory neuronal effect, it is essential to perform further studies combining fMRI and electrophysiological techniques. Second, whether it is opportune to low-pass filter the resting-state fMRI data for GCA is still unclear. Although previous studies give possible and rational reasons for using the filtered [56] or non-filtered [40] fMRI data, a comprehensive investigation is necessary to measure the contribution of different subfrequencies. Further studies are needed to clarify this meaningful point. Finally, removing or keeping global signal would affect the functional connectivity during the resting state [64]. This influence in GCA remains unclear. But because an identical processing procedure was used in both groups, the between-group differences bias was minimized.

In addition, two potential confoundings should be noted. First, since our study did not simultaneously record EEG with fMRI data, it remains unclear whether the abnormal causal effects in patients related to the interictal epileptic discharges. Second, the anti-epileptic drugs may influence brain functions [65], although the patients had discontinued medication for about 24 h before scanning. Future studies on drug naïve patients may make the understanding of pathophysiological mechanism of mTLE more clearly.

## Conclusions

In the current study, we characterized the causal relation between EZ and the whole brain using ALFF and GCA. Compared with that of controls, EZ showed an abnormal causal relation with thalamus, BG, and several cortical networks, suggesting its abnormal causal interaction with widespread brain regions. The correlation between the GCA value and disease duration suggested the causal effect may reflect the progress of mTLE. Furthermore, the local activity level in EZ was positively correlated with the abnormal driving effect on it, suggesting the initiation of epileptic activity depends not only on EZ itself, but also on the activity emerging in large-scale macroscopic brain networks. Overall, the current study found that the causal topological organization is disrupted in mTLE, providing valuable information to understand the pathophysiology of this disorder.

## Materials and Methods

### Participants

This study involved twenty-three patients with left mTLE (all right-handed, 12 female; ages, 27.0±8.5 years; epilepsy durations, 10.8±8.1 years). All patients underwent a comprehensive clinical evaluation and met the following diagnostic criteria: (i) Symptoms of mTLE: all patients had complex partial seizures, accompanied, or not, by secondary generalized seizures or simple partial seizures. Each patient presented one or more typical symptoms of mTLE, such as abnormal emotional experiences and psychiatric symptoms, epigastric rising, automatisms, and dystonic posturing of the limbs. (ii) MRI manifestation of unilateral hippocampal sclerosis: Hippocampal volume less than the hippocampal volume in healthy Chinese (2.48 cm^3^ on the left) as measured in coronal T1 images and an increase in T2 fluid-attenuated inversion recovery signal in the hippocampus were used as diagnostic criteria [66]. There was no other MRI abnormality than the hippocampal sclerosis. (iii) Electroencephalography findings: Interictal and ictal scalp electroencephalography showed epileptic spikes in the left frontotemporal/temporal lobes (FPS, F7/F8, T3/T4 and T5/T6) in the patients.

Twenty-three age- and sex-matched healthy volunteers (right-handed) were recruited as controls (age, 25.7±8.7 years). None of them had neurological or psychiatric disorders.

All examinations were carried out under the guidance of the Declaration of Helsinki (1975). Written informed consent forms were obtained from all the groups. The research protocol was approved by the local Medical Ethics Committee of Jinling Hospital, Nanjing University School of Medicine.

### Data Acquisition

We performed functional and structural neuroimaging scanning for mTLE patients and normal controls using a Siemens Trio 3T scanner at Jinling Hospital. We used foam padding to minimize head motion. We acquired resting-state functional images using a single-shot, gradient-recalled echo planar imaging sequence (250 volumes, repetition time = 2000 ms, echo time = 30 ms, flip angle = 90°, field of view = 240×240 mm^2^, interslice gap = 0.4 mm, voxel size = 3.75×3.75×4 mm^3^, 30 transverse slices aligned along the anterior–posterior commissure). We instructed subjects simply to rest with their eyes closed, not to think of anything in particular, and not to fall asleep. Subsequently, we acquired high-resolution T1-weighted anatomical images in sagittal orientation using a magnetization-prepared rapid gradient-echo sequence (repetition time = 2300 ms, echo time = 2.98 ms, flip angle = 9°, field of view = 256×256 mm^2^, voxel size = 0.5×0.5×1 mm^3^, 176 slices without interslice gap).

### Functional magnetic resonance imaging data preprocessing

Functional image preprocessing was carried out using the DPARSF (http://www.restfmri.net) [67] and SPM8 (http://www.fil.ion.ucl.ac.uk/spm) toolkits. The first 10 functional volumes were discarded as signal equilibrium and subjects' adaptation to scanning noise. We corrected the remaining images for temporal differences and head motion. No translation or rotation parameters in any given data set exceeded ±2 mm or ±2°. We warped the functional images into a standard stereotaxic space at a 3×3×3 mm^3^ resolution, using the Montreal Neurological Institute (MNI) echo-planar imaging template, and then we spatially smoothed them with a 4-mm full-width half-maximum (FWHM) isotropic Gaussian kernel. Finally, we removed linear trends from time courses and for temporal band-pass filtering (0.01–0.08 Hz) [56], [68].

### ALFF analysis

For amplitude measures at each voxel, we used ALFF [13]. The ALFF was defined as the averaged square root of activity in the low-frequency band (0.01–0.08 Hz). The ALFF value of each voxel was standardized by dividing the full-brain mean ALFF values.

The two-sample t-tests were employed to compare the differences in ALFF between groups. Using the REST AlphaSim program, a corrected significance level of P<0.05 was obtained by clusters with a minimum volume of 1458 mm^3^ and individual voxel height threshold of P<0.05.

### Granger causality analysis

From the resulting map of the between-group analysis of ALFF, peak voxel within left mTL was selected as a sphere seed with a radius of 3 mm for the following GCA [33], [69]. The left mTL was defined by including the left hippocampus, para-hippocampus, and amygdala from the automatic anatomical labeling template [70]. The voxel-wise coefficient GCA [33] was performed in the whole brain using REST-GCA, a plug-in implemented in REST software (http://www.restfmri.net) [69]. GCA was first proposed for determining whether the past value of a time course could correctly forecast the current value of another. If the current value of time course Y could be more accurately estimated by the combination of past value of time courses X and Y than the past value of Y alone, then X has Granger causal influence on Y. Granger causality is often estimated using vector autoregressive models. Coefficient-based GCA used the regression coefficient β in vector autoregressive models to estimate Granger influence [33], [40], [69]. A positive value of β may indicate positive influence, and a negative β may indicate inhibitory influence. In the current study, we applied bivariate coefficient GCA to investigate the causal relation between EZ and each voxel in the entire brain. For the seed(EZ)-to-whole-brain, one-sample t-tests were performed for the causal effects within each group with an AlphaSim-corrected significance level of P<0.05 (height threshold, P<0.05; extent threshold, k = 1458 mm^3^). The resulting maps of the two groups were combined and taken as a “causal effect mask”. Two-sample t-tests were performed on the causal effects between groups within the “causal effect mask” with an AlphaSim-corrected significance level of P<0.05 (height threshold, P<0.001; extent threshold k = 135 mm^3^). The analysis for whole-brain-to-seed(EZ) was performed in the same way as the seed(EZ)-to-whole-brain.

To explore whether the causal effect correlates with the disease progression in mTLE patients, a Pearson correlation analysis between causal effect and disease duration was performed at the peak voxel of clusters from the between-group analysis. To explore the relationship between ALFF and causal effect, we averaged the causality values of peak voxels in clusters showing abnormal driving effect on EZ and correlated it with the ALFF value of EZ in each group, using Pearson correlation.
