# Correction: Disrupted Causal Connectivity in Mesial Temporal Lobe Epilepsy

**DOI:** 10.1371/annotation/af51084c-0a13-4848-8a6c-9c33e757fd0e

**Published:** 2013-09-10

**Authors:** Gong-Jun Ji, Zhiqiang Zhang, Han Zhang, Jue Wang, Dong-Qiang Liu, Yu-Feng Zang, Wei Liao, Guangming Lu

The number of the grant from the Natural Science Foundation of China to the sixth author is incorrect. The correct grant number is: 81020108022. 

